# Research on the Common Fevers

**Published:** 1921-04-30

**Authors:** 


### RESEARCH ON THE COMMON FEVERS.

### By a Medical Officer of Health.

Colonel Sir William Smith, at a recent meeting of the Metropolitan Asylums Board, moved the following resolution: "That the Ministry of Health be informed that, in the opinion of this Board, the time has now arrived when the scientific inquiry into the causation, infectivity, prevention, and treatment of zymotic disease, which was instituted by the Board in 1913, should be taken over by the Committee of the Privy Council for Medical Research (Medical Research Council), and that the Ministry be asked whether they will be prepared to make the necessary arrangements with a view to this being done."

In moving this resolution Sir William has given us all a timely reminder that there is still an enormous amount of research on these diseases still to be done, a research which cannot possibly be done by anyone who has not at his or her disposal the material existing in the British Isles as a whole, and, if necessary, that which exists in other countries as well.

### Larger Resources Needed.

It is well known to epidemiologists that the behaviour of these diseases presents enormous differences in different parts of the British Isles, as well as in different countries, differences due to climate, constitution of the population, their prevalence among different peoples, differences due to habits, natural immunity, the existence of different kinds of insects which often act as intermediate hosts, and a number of other causes which would take too long to enumerate. It is therefore quite obvious that such a work can only be effectively undertaken by a body with very large resources at its command.

That such a research is imperative in the interests of the health of the community, as well as of their pockets, will be realised on a consideration of a few of the facts connected with our attempts to prevent and treat even the best-known fevers.

In the first place, we are quite ignorant of the causal germ in many common zymotics, notably smallpox, scarlet fever, measles, whooping-cough, typhus fever, poliomyelitis, and influenza, to mention only a few of the more important and deadly ones. It is also true that we know the morbific agent in diphtheria, enteric fever, cerebro-spinal meningitis, and a few others, but in none of these, with the possible exception of malaria, do we know the complete life history of the agents and why in some cases, such as enteric fever, they may appear to be dying a natural death, and yet in these and others they can become suddenly more virulent and attain a power of spreading through the community. Witness the recent pandemics of influenza and the epidemics of typhus on the Continent. Various explanations have been put forward from time to time to account for these events, such as increased susceptibility of the population due to war stress, increased virulence of the bacteria from unknown causes, but so far no explanation will fit in with all the facts, either generally or individually.

The second point is that our present efforts have notably failed to stem the prevalences of many common fevers, such as scarlet fever, diphtheria, measles, and whooping-cough. Some twenty-five years ago isolation hospitals, coupled with thorough disinfection, were hailed as the panacea for scarlet fever and diphtheria; but, in spite of the most ample provision, especially in London, these diseases seem to be as prevalent as ever, and it is therefore high time that a searching inquiry be made into our present methods.

### Where Treatment Stands.

In regard to treatment we are still more in the dark, for in only one disease—diphtheria—have we discovered a specific cure in anti-toxin, a cure which is as nearly infallible as any cure is ever likely to be; but, while we have been able to make use of our knowledge of the diphtheria bacillus to effect this revolution in the treatment, we are, unfortunately, not in a position to make use of this knowledge to make a vaccine which can be easily and generally used to prevent the occurrence of the disease. There are only two diseases which we really have power to prevent completely, and these are smallpox and malaria, by the full use of vaccination and killing of mosquitoes. Even in enteric fever, though we can produce a temporary immunity, we have failed to extend this immunity beyond a couple of years.

We trust, therefore, that the Ministry will seriously consider this proposal, with a view to the research into these diseases being begun at the earliest possible moment.

